# Identification of Spike Length Gene and Development of KASP Markers in Wheat

**DOI:** 10.3390/plants14233703

**Published:** 2025-12-04

**Authors:** Tiantian Jiang, Lingpeng Meng, Chao Ji, Zehui Wang, Huiwen Cao, Ruoxi Sun, Ke Xu, Xianghai Meng, Xueju Yang, Yong Zhao

**Affiliations:** 1 State Key Laboratory of North China Crop Improvement and Regulation, Hebei Agricultural University, Baoding 071000, China; jiangtiant2025@163.com (T.J.); menglingp2025@163.com (L.M.); jichao181@163.com (C.J.); wangzehuuu@163.com (Z.W.); caohuiwen2020@163.com (H.C.); sunrxhebei@163.com (R.S.); xuke@hebau.edu.cn (K.X.); 2 Key Laboratory of Crop Germplasm Resources in North China, College of Agronomy, Hebei Agricultural University, Baoding 071000, China; 3 Dry Farming Institute (DFI), Hebei Academy of Agricultural and Forestry Sciences (HAAFS), Hengshui 053000, China; mengxianghai5229@163.com

**Keywords:** wheat (*Triticum aestivum* L.), long spike mutant, BSA-seq, gene mapping, marker development

## Abstract

Spike length is a critical trait influencing the yield potential of wheat (*Triticum aestivum* L.). However, there has been limited research on spike-length-related genes in wheat. Moreover, the scarcity of stable markers for spike-related traits has restricted marker-assisted selection-based breeding. In this study, a novel long-spike mutant material (*LS1*) was generated from wheat variety ‘Aikang 58’ (AK58) using ethyl methanesulfonate. We established an F_2_ segregating population by crossing AK58 with *LS1*. Morphological analyses of this population indicated that spike length is a dominant quantitative trait regulated by multiple genes. Bulked segregant analysis (BSA) technology was used to preliminarily identify nine candidate regions associated with spike length traits. These regions were mainly in a 7.22 Mb interval (673.84–713.26 Mb) on chromosome 5A and in a 2.34 Mb interval (714.83–717.69 Mb) on chromosome 7B. Twelve candidate genes were identified within these regions. Furthermore, two kompetitive allele specific polymerase chain reaction (KASP) markers (*KASP-LS1-681460621* and *KASP-LS1-692013966*) associated with spike length traits were developed. Both KASP markers effectively genotyped parental lines and the F_2_ population. Our study results provide a theoretical foundation for the genetic improvement of spike-length-related traits in wheat.

## 1. Introduction

Wheat (*Triticum aestivum* L.), which is a critical food crop, is cultivated worldwide and serves as a primary dietary component for billions of people [1]. To continue to feed the growing global population and maintain agricultural profitability, wheat production must increase, even with decreasing wheat cultivation areas [2]. Hence, wheat yield potential must be optimized. As one of the yield-related traits of wheat, spike length affects the number of spikelets and grains per spike [3]. Long spikes usually have more spikelets and grains than short spikes. Thus, increasing spike length can effectively increase individual spike yields without changing spikelet density [4,5]. Chinese varieties with long and rectangular spikes are popular among farmers and seed distributors because of their exceptional field performance and commercial viability, leading to their cultivation across vast regions. Exploring genetic loci related to spike length and analyzing the molecular mechanism underlying spike elongation may be conducive to increasing wheat yields, while also enhancing molecular marker-assisted breeding.

Although numerous quantitative trait loci (QTLs) for spike length have been identified across all 21 wheat chromosomes [6,7,8], the associated research remains largely at the QTL mapping stage. For example, nine QTLs explaining 9.74–22.29% of the phenotypic variation were identified on chromosomes 1A, 1B, 1D, 2D, 4A, 5A, and 5D using recombinant inbred line (RIL) populations (WL711 × PH132 and Opata85 × W7984) [9]. Similarly, five major QTLs for spike length (explaining 7.08–16.35% of the phenotypic variation) were detected on chromosomes 3B, 4A, and 7A in an RIL population (Bhalegaon × 4PDW233) [10]. Using a wheat RIL population (TAA10 × XX329), Xu et al. detected seven QTLs related to spike length (explaining 7.22–11.59% of the phenotypic variation) in the D subgenome [11]. In another study, QTLs significantly related to spike length (explaining 8.8–36.6% of the phenotypic variation) were mapped on chromosomes 4A and 7B using F_2_ and F_2:3_ populations (10 A × BE89) [12]. On the basis of an F_2_ population and RIL population (Nanda2419 × Wangshuibai), Ma et al. detected a QTL controlling spike length (explaining 22.6% and 31.4% of the phenotypic variation, respectively) on chromosome 7D [13]. Using a wheat F_2:3_ population (Z559 × Fusuimai) comprising 237 lines, QTLs for spike length were identified on chromosomes 2DS, 3B, 4AL, 5A, 6AL, and 6BL [14]. However, because of differences in the conclusions drawn from different mapping populations, most of these QTLs are associated with low repeatability and large confidence intervals. Moreover, only a few major QTLs have been identified, and the genetic basis of spike elongation remains unclear, which has limited the utility of these QTLs for improving wheat production. Therefore, spike-length-related genes must be identified and the genetic mechanisms related to spike length should be further analyzed to improve wheat grain yields.

A bulked segregant analysis (BSA) involving the construction of mixed pools of samples with extreme phenotypes is useful for rapidly identifying regions associated with target traits [15,16]. In addition, BSA-seq, which combines BSA and exon capture sequencing, is mainly used for gene localization or cloning [17]. To date, it has been widely used for mapping wheat spike-length-related genes. For example, a previous study used BSA technology and the Wheat 660K chip analysis of 262 BC_1_F_7_ lines (Chuanyu25 × Chuanyu12D7) to map a QTL controlling wheat spike length and spike density to a 36.89 cM interval on chromosome 2D [18]. Additionally, through BSA-seq analysis of a wheat RIL population (13F10 × Chuanmai42), Ji et al. determined that the spike length trait was controlled by a 6.69 Mb region (518.43–525.12 Mb) on chromosome 5AL [19]. On the basis of an F_2_ population (Pubing3228 × Jing4839), 25 candidate regions controlling grain number per spike were mapped using BSA and SLAF-seq techniques, with 399 genes within these regions [20]. In another study, an RIL population (BLM × CY20) was constructed and BSA-seq technology was used to map three genomic regions associated with spike length on chromosomes 2A and 2D [21]. Therefore, BSA-seq technology is effective for mining target genes related to wheat spike length traits. Creating materials with mutated spike-length-related traits and exploring new gene loci controlling spike length may lead to new approaches to improving agronomic and yield-related traits of wheat.

In this study, a long-spike mutant, *LS1*, which was derived from wild-type (WT) ‘Aikang 58’ (AK58) via ethyl methanesulfonate (EMS) mutagenesis, was crossed with AK58 to produce the F_1_ generation. The subsequent genetic analysis of the F_1_ generation was performed to determine the dominance and recessiveness of spike length traits. An F_2_ segregating population was established by self-pollinating the F_1_ generation. Furthermore, candidate genes were predicted via BSA-seq, while kompetitive allele specific polymerase chain reaction (KASP) markers were developed and validated. The study results provide a theoretical foundation for the genetic improvement of traits associated with spike length in wheat.

## 2. Results

### 2.1. Phenotypic Identification and Genetic Analysis

**Figure 1:**
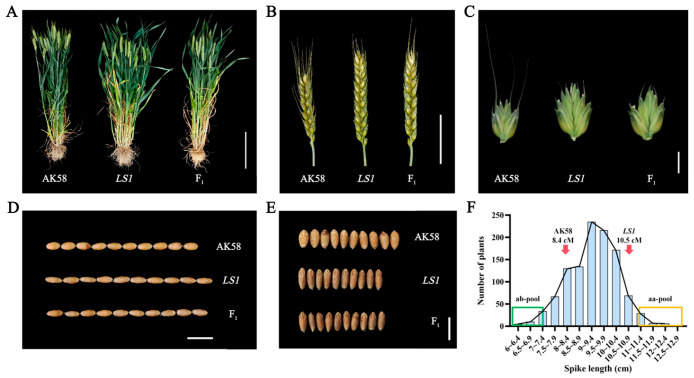
Phenotypic characteristics of wild-type AK58, mutant *LS1*, and F_1_. (**A**) Plant height of AK58, *LS1*, and F_1_, scale = 20 cm; (**B**) Spike length of AK58, *LS1*, and F_1_, scale = 5cm; Spikelet (**C**), grain width (**D**), and grain length (**E**) of AK58, *LS1*, and F_1_, scale = 1 cm; (**F**) Distribution of spike length in the F_2_ segregating population (*n* = 1120).

Several phenotypic traits differed significantly among AK58 (i.e., WT control), *LS1*, and F_1_ plants. A comparison with WT plants revealed a 9.71% decrease in the height of *LS1* plants (Figure 1A and Figure S1A). By contrast, spike length increased by 29.05% (Figure 1B and Figure S1B), spikelets increased in size, and the number of spikelets increased by 13.80% (Figure 1C and Figure S1C). Additionally, the number of grains per spike increased by 36.84% (Figure S1D), while grain length increased by 7.16% (Figure 1D and Figure S1E). However, grain width decreased by 11.24% (Figure 1E and Figure S1F). The number of effective spikes increased by 27.92% (Figure S1G), while the yield per plant increased by 35.14% (Figure S1H). F_1_ plants resulting from the hybridization between *LS1* and AK58 grew normally. Field observations indicated that all F_1_ plants had long spikes, reflecting the dominance of this trait (Figure 1B). In addition, the spike length data for 1120 independent F_2_ plants were normally distributed (Figure 1F). Furthermore, according to an analysis of the F_2_ segregating population (AK58 × *LS1*), spike length varied considerably (6–12.90 cm). Hence, the spike length trait of the two parents is a typical quantitative trait, making it suitable for QTL mapping.

### 2.2. BSA-Seq Analysis

Using the MGI-2000/MGI-T7 sequencing platform with a PE150 sequencing strategy, 83.64 Gb raw sequencing data were obtained by sequencing the two parents and two extreme bulks. After filtering, 83.26 Gb high-quality data remained (Table 1). Quality assessment revealed that the sequencing data were high quality (Q20 ≥ 98.86% and Q30 ≥ 96.84%). When the Chinese Spring reference genome was used, the alignment success rate was greater than 99.71% and the coverage rate exceeded 96.88%, with an average sequencing depth of 49.63 and 57.21 for the parent and offspring pools, respectively. Accordingly, the sequencing data were highly reliable and suitable for detecting mutations and analyzing correlations.

**Table 1:** The quality summary of BSA-Seq data.

Sample	Aikang 58	Long Spike Mutants	Normal Offspring Mixed Pool	Long Spike Offspring Pool
Raw Reads Number	133,225,654	124,622,072	141,320,642	158,423,946
Clean Reads Number	133,225,650	124,622,072	141,320,642	158,423,946
Raw Bases (bp)	19,983,848,100	18,693,310,800	21,198,096,300	23,763,591,900
Clean Bases (bp)	19,903,818,088	18,614,137,400	21,098,284,286	23,641,511,070
Effective Rate (%)	99.60	99.58	99.53	99.49
Q20 (%)	98.86	98.86	98.88	98.90
Q30 (%)	96.86	96.84	96.90	96.98
Align reads number	132,849,145	124,431,656	140,910,807	158,167,511
Align rate (%)	99.72	99.85	99.71	99.84
Target region size (Mb)	236.64	237.91	232.73	231.11
Target region coverage Rate (%)	96.88	97.53	98.56	98.58
Depth (X)	50.09	49.17	53.26	61.15

Note: Effective rate: the proportion of the clean sequence reads mapped to the reference genome relative to the total clean reads; Q20: Percentage of bases correctly identified above 99%; Q30: Percentage of bases correctly identified above 99.9%.

### 2.3. Mutation Site Detection

**Figure 2:**
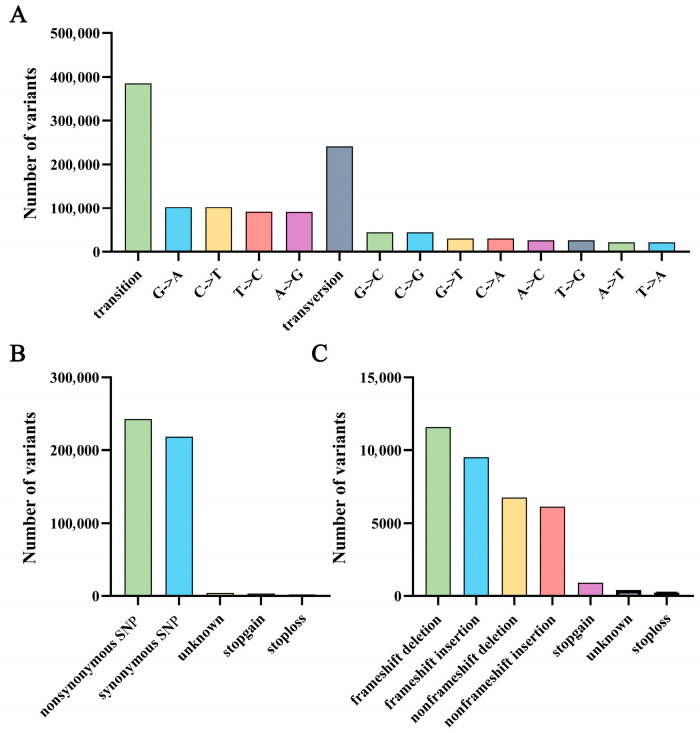
Detection of variant sites. (**A**) SNPs type statistics table; (**B**) SNPs function annotation statistics in the CDS region; (**C**) InDels function annotation statistics in the CDS area.

After SNP and InDel detection, a total of 715,679 variations were screened from the four DNA pools. Among these variation sites, 626,506 were SNPs and 89,173 were InDels. Among the SNPs, 385,336 were transition types, and 241,170 were transposable types (Figure 2A). After excluding sites with a coverage depth of less than 5× using a Perl script, 533,870 effective SNPs were obtained. Including 243,012 non-synonymous mutations, 218,341 synonymous mutations, 4086 unknown mutations, 3343 stop-gain mutations, and 672 stop-loss mutations (Figure 2B). Among the InDels, most of these mutation sites were concentrated in intron and exon regions (CDS, Table 2). The mutations in exon regions included 6759 non-frameshift deletions, 11,604 frameshift deletions, 6140 non-frameshift insertions, 9514 frameshift insertions, 902 stop-gain mutations, 74 stop-loss mutations, and 400 unknown mutations (Figure 2C).

### 2.4. SNP-Index Association Analysis

A total of 160,586 SNPs were retained by filtering 533,870 SNPs. Subsequently, significant differences in genotype frequency between mixed pools were assessed on the basis of Δ(SNP-index), which revealed 152,425 SNPs with an offspring SNP-index less than 0.30. After calculating Δ(SNP-index), the distribution of Δ(SNP-index) on the chromosomes of the two extreme-phenotype offspring pools was mapped (Figure S2A). On the basis of a positive threshold (0.95), 213 candidate regions comprising 259,500,213 bp were identified (Table S1). In these regions, 109 loci were distributed on chromosomes 2A, 2B, 3A, 4B, 5A, 6B, 7A, and 7B. Interestingly, 47 of these loci (45% of all identified loci) were on chromosome 5A (Figure S2B). According to their physical positions, 57 genes were associated with these SNPs (Table S2).

### 2.5. Euclidean Distance Analysis

During the Euclidean distance (ED) analysis, 479,133 loci were identified after filtering (Figure S3A). Using the association threshold (median + 3SD of all site fitted values, 0.0567), 10 candidate regions were obtained (Table 3), with a total length of 11,323,701 bp, after which candidate SNP loci were selected from these regions based on two criteria: main-allele frequency > 0.75 in the mutant pool and ED > 0.50. A total of 189 SNP loci were selected (Table S3). These loci were mainly on chromosome 5A (141 loci; approximately 75% of all identified loci), but some were on chromosome 7B (Figure S3B). The ANNOVAR annotation results for candidate SNP loci revealed the potential involvement of 71 genes.

### 2.6. Target Trait Region Mapping

On the basis of the annotated SNPs, the regions identified by two association analyses were combined, resulting in the mapping of nine regions on chromosomes 5A (673.84–713.26 Mb) and 7B (714.83–717.69 Mb), with a total length of 9.56 Mb (Table 4). These nine regions included 46 candidate SNPs, with 43 SNPs on chromosome 5A and three SNPs on chromosome 7B. These loci included 13 non-synonymous mutations, 13 synonymous mutations, and 2 unknown mutations in exons. Additionally, four SNPs were in the 3′ untranslated region (UTR3), one was in the 5′ untranslated region (UTR5), one was in 2 kb upstream and downstream regions, and 12 were in introns (Table S4).

### 2.7. Candidate Gene Prediction and Expression Analysis

**Figure 3:**
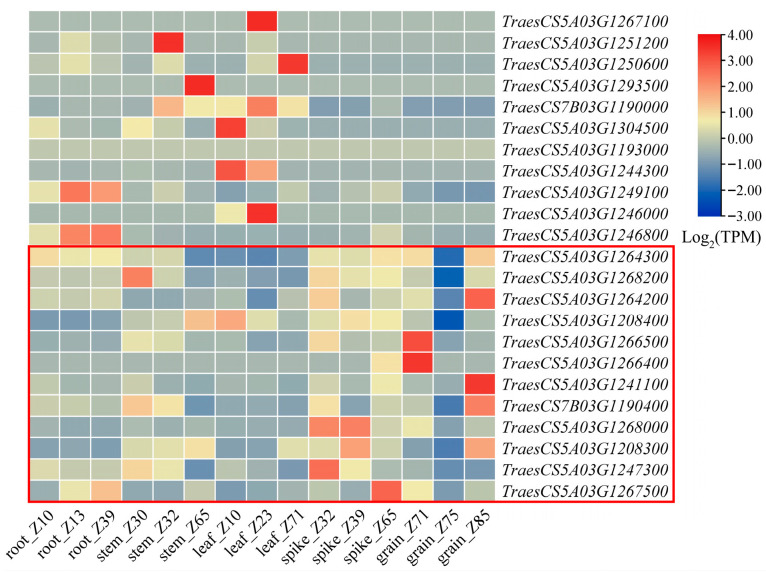
Temporal and spatial expression patterns of 23 candidate genes. Twelve genes expressed in spike were marked by red boxes.

A total of 23 candidate genes were identified according to their positions in the reference genome and the corresponding gene location information for candidate SNP loci (Table 5). ANNOVAR software (version 2022-01-13) was used to functionally annotate these genes. The annotated genes included those encoding the following: protein with a protein kinase domain (*TraesCS5A03G1208300*), PAIR1 (*TraesCS5A03G1241100*), tubulin (*TraesCS5A03G1247300*), GRAS transcription factor (*TraesCS5A03G1249100*), aspartate peptidase A1 family member (*TraesCS5A03G1266400*, *TraesCS5A03G1266500*), and leucine-rich repeat domain superfamily member (*TraesCS5A03G1267500*). Our analysis of the tissue-specific expression of the 23 candidate genes using available RNA-seq data indicated that 12 genes were predominantly expressed in the wheat spike (Figure 3). Of these 12 genes, the following four were highly expressed in the spike: *TraesCS5A03G1268000*, *TraesCS5A03G1208300*, *TraesCS5A03G1247300*, and *TraesCS5A03G1267500*.

### 2.8. KASP Marker Development and Validation

**Figure 4:**
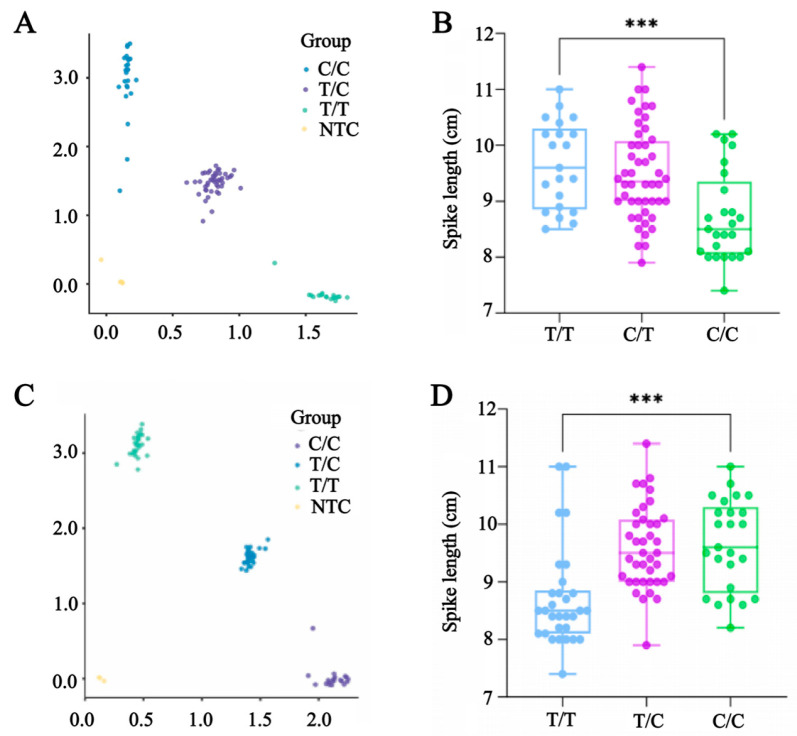
Genotyping of KASP markers in parents, bulk pools, and F_2_ population. (**A**) Genotyping of marker *KASP-LS1-68146062* in parents and bulk pools. (**B**) Genotyping of marker *KASP-LS1-681460621* in F_2_ population. (**C**) Genotyping of marker *KASP-LS1-692013966* in parents and bulk pools. (**D**) Genotyping of marker *KASP-LS1-692013966* in F_2_ population. *** *p* < 0.001.

Mapping results were used to develop six KASP markers for the high-quality SNP loci associated with 12 candidate genes (Table S5). KASP genotyping of 91 randomly selected F_2_ population DNA samples, combined with spike length data, was used to assess marker utility. The results indicated that two KASP markers (*KASP-LS1-681460621* and *KASP-LS1-692013966*) were useful for genotyping the F_2_ population, with genotypes largely aligning with phenotypes (Figure 4). For *KASP-LS1-681460621*, TT, CC, and CT genotypes corresponded to the long-spike group, short-spike group, and heterozygous group, respectively (Figure 4A). A *t*-test revealed a significant difference in spike length between the long-spike group and the short-spike group (*p* = 0.0002) (Figure 4B). For *KASP-LS1-692013966*, CC, TT, and TC genotypes corresponded to the long-spike group, short-spike group, and heterozygous group, respectively (Figure 4C). A *t*-test for *KASP-LS1-692013966* confirmed that the long-spike group and short-spike group differed significantly in terms of spike length (*p* = 0.0001) (Figure 4D).

## 3. Discussion

### 3.1. Identification of Key Spike-Length-Related Genetic Loci Relevant to Increasing Wheat Yields

Identifying wheat yield-related genes or QTLs is critical for characterizing yield formation and optimizing grain production via genetic improvement [22]. Spike length represents a key quantitative trait in this context, with a complex genetic basis (i.e., numerous genes and factors). Germplasm resources with long spikes, which are characterized by an elongated spike axis and many spikelets, may be used to increase spike fertility and grain number, thereby increasing grain yields [23]. Our analysis of the long-spike mutant *LS1* revealed a significant increase in flag leaf size (42.19% increase in length and 22.22% increase in width) as well as substantial increases in spike length (29.05%) and grain number per spike (36.84%), ultimately resulting in increased yield per plant (Figure 1 and Figure S1). To identify causal loci for these phenotypic changes, we conducted a BSA-seq analysis. Integrating this approach with other methods involving wheat genome sequencing technologies and modern SNP platforms is indispensable for a comprehensive understanding of the genetic basis of spike elongation and for optimizing wheat yields [24,25].

### 3.2. Analysis of Wheat Spike-Length-Related Candidate Genomic Regions

The genomic regions associated with spike length on chromosome 5A were identified in previous studies [19,26,27,28]. Zhai et al. used an RIL population comprising 191 F_9_ materials derived from the cross between ‘Yumai 8679’ and ‘Jing 411’ to map *QSl.cau-5A.4*, which regulates spike length, to the 510.51–538.76 Mb interval on chromosome 5AL [26]. Similarly, Hu et al. used Chinese variety ‘Yanzhan 1’ as a common parent for hybridizations with four donor parents (i.e., British variety ‘Hussar’ and three Chinese wheat varieties), thereby constructing four RIL populations, after which six QTLs controlling spike length were mapped to the 381.76–533.29 Mb interval on chromosome 5AL [27]. Ji et al. localized *QSl.cib-5A*, which regulates spike length, to a 6.69 Mb region (518.43–525.12 Mb) on chromosome 5AL following a BSA-seq analysis and then developed KASP and SSR markers; this region was subsequently narrowed to 4.84 cM, corresponding to a 4.67 Mb physical region (516.60–521.27 Mb) [19]. The primary loci associated with wheat spike length identified in the present study were, respectively, mapped to the 673.84–713.26 Mb interval on chromosome 5A (i.e., considerable distance from the aforementioned intervals; Table 4). In addition, a novel locus on chromosome 7B was identified, which has not been reported in previous studies focused on spike length.

Zhang et al. identified the genomic locus responsible for spike type within the 642.9–706.1 Mb region on chromosome 5A and identified *Q* (650.127–650.130 Mb) as a potential candidate gene [28]. By contrast, our study revealed that the region associated with spike-related traits overlaps the region identified by Zhang et al. [28] but does not include the *Q* locus. This suggests that novel loci are present in this non-overlapping region, as well as within the newly identified region on chromosome 7B, which will need to be more thoroughly investigated in future studies.

### 3.3. Candidate Gene Expression and Function

Numerous researchers have identified and characterized multiple genes associated with spike length [29,30,31,32,33]. In the present study, 12 candidate genes related to spike length were identified, among which 4 were differentially expressed at different spike developmental stages (Figure 3). *TraesCS5A03G1267500* encodes a protein with a leucine-rich repeat domain (20–29 residue motif present in functionally diverse proteins), which primarily serves as a versatile structural scaffold for protein–protein interactions [34,35]. *TraesCS5A03G1247300* is a tubulin gene (designated as *TaTUB-5A5* in the *TaTUB* family) identified by Ren et al. [36]. Notably, the *TaTUB-5A5* promoter has many light-responsive cis-acting elements, implying that the encoded protein affects plant photomorphogenesis. *TraesCS5A03G1268000* encodes an E3 ubiquitin ligase known to be involved in flowering, cell cycle progression, and spike inflorescence development [37,38,39,40]. Importantly, some E3 ubiquitin ligase family members have been shown to positively regulate spike elongation [41,42]. *TraesCS5A03G1208300* is characterized by a sequence encoding a protein kinase domain. Protein kinases phosphorylate proteins, serving as critical regulatory switches for cell growth and proliferation [43]. While the molecular mechanisms and signaling pathways involving these genes remain to be fully elucidated, the identification of these candidates provides a foundation for dissecting the genetic mechanisms controlling spike length.

### 3.4. Development of KASP Markers for Long Spikes and Screening of Effective Genotyping Markers

There are relatively few stable markers for spike-related traits useful for marker-assisted selection-based breeding, although numerous loci have been identified in previous studies [44,45]. This may be related to the high sensitivity of spike-related traits to environmental factors as well as the considerable distance between markers and target genes [46]. To develop KASP markers applicable for high-throughput genotyping, SNPs significantly associated with spike length identified via BSA-seq were selected, after which KASP assays were conducted. Of the six KASP markers developed in this study, *KASP-LS1-681460621* and *KASP-LS1-692013966* were useful for genotyping parental and F_2_ populations (Figure 4). The genotypic and phenotypic profiles were largely consistent, enabling long-spike and short-spike genotypes to be distinguished. Using these markers may substantially accelerate the selection of favorable alleles in wheat breeding programs and improve the accuracy of genetic mapping.

## 4. Materials and Methods

### 4.1. Plant Materials and Phenotypic Data Collection

In the summer of 2018, a long-spike mutant (*LS1*) was identified in the EMS mutant library of wheat variety AK58, which was developed in our laboratory. Notable features of this mutant were as follows: increased spike length, increased grain number per spike, and rectangular spike. *LS1* was continuously self-pollinated until its traits stabilized. In October 2023, *LS1*, AK58, and two hybrid generations (F_1_ and F_2_) were grown in the experimental field of the Crop Breeding Center at Hebei Agricultural University (38°83′ N, 115°45′ E), with 10 cm between plants and 23 cm between rows. Local field management practices were applied. The F_2_ segregating population consisted of approximately 1120 individual plants. At physiological maturity, 20 representative AK58, *LS1*, and F_1_ generation plants were harvested to examine the following agronomic traits: plant height, effective tiller number per plant, spike length, flag leaf length and width, grain number per spike, spikelet number, and thousand-grain weight.

### 4.2. BSA-Seq

#### 4.2.1. DNA Extraction and Mixed Pool Construction

On the basis of the phenotypic analysis of the F_2_ population, two DNA pools were constructed by mixing equal amounts of DNA from 30 long-spike individuals (aa pool) and 30 short-spike individuals (ab pool). Additionally, we selected 10 plants from each parental line to form a parent pool. Genomic DNA samples extracted using the CTAB method [47] were placed in 1.5 mL centrifuge tubes stored in dry ice and sent to Molbreeding Biotechnology Co., Ltd. (Shi Jiazhuang, China) for database construction and sequencing.

#### 4.2.2. Reference Genome Alignment and SNP Detection

After sequencing, raw data were filtered for quality using the sliding window method and fastp software (version 1.0.1) [48]. Specifically, clean reads were obtained by discarding paired reads in which low-quality bases (Q < 20) exceeded 40% of the read length and the number of N bases was greater than 10. Clean reads were aligned to the Chinese Spring reference genome sequence (IWGSC RefSeq v2.1) using BWA-MEM software (version 0.7.19, mem alignment method) to determine their physical locations [49]. SNPs and InDels were called and filtered by removing heterozygous and missing SNPs and InDels in the pools and parental lines using GATK software (version 4.0) [50], with the identified SNPs annotated using ANNOVAR software (version 2022-01-13) [51]. Candidate genes were identified using the SNP-index association algorithm and the ED association algorithm, with the results subsequently combined. The SNP-index method relies on differences in genotype frequencies among pools to identify significant deviations in genotype frequencies within pools. The following formulae were used:

$$SNP\;index\left(aa\right)=Maa/(Maa+Paa),$$

with Maa representing the depth of the aa pool from the female parent and Paa representing the depth of the aa pool from the other parent.

$$SNP\;index\left(ab\right)=Mab/(Mab+Pab),$$

with Mab representing the depth of the ab pool from the female parent and Pab representing the depth of the ab pool from the other parent.

$$\Delta SNP\;index=SNP\;index\left(aa\right)-SNP\;index(ab)$$

Unlike the SNP-index method, the ED association algorithm uses sequencing data and identifies markers that differ significantly between pools, thereby facilitating the assessment of associations between genomic regions and target traits.

$$ED=\sqrt{{\left(A_{mut}-A_{wt}\right)}^{2}+{\left(C_{mut}-C_{wt}\right)}^{2}+{\left(G_{mut}-G_{wt}\right)}^{2}+{\left(T_{mut}-T_{wt}\right)}^{2},}$$

with (*A*, *T*, *C*, and *G*)*_mut_* representing the frequency of the mutant mixed-pool bases and (*A*, *T*, *C*, and *G*)_wt_ representing the frequency of the WT mixed-pool bases.

### 4.3. Candidate Region Analysis and Gene Identification

All genes identified in the associated regions were annotated using ANNOVAR software (version 2022-01-13) and the Chinese Spring reference genome (IWGSC RefSeq v2.1). Candidate gene expression patterns in various wheat growth stages and tissues were analyzed using the Chinese Spring Gene Expression Database (http://202.194.139.32 (accessed on 14 February 2024)).

### 4.4. KASP Marker Development

After downloading the sequences of 150 bp fragments upstream and downstream of SNP loci from the Wheat Omics 1.0 database, their specificity and utility for developing molecular markers associated with long spikes were evaluated. SNP loci with fewer than three copies and a GC content of 40–60% were retained. KASP marker primers were designed using DNAMAN software (version 7.0). Each primer mix consisted of two specific forward primers and one universal reverse primer (19–30 nucleotides long). The two forward primers differed only at the terminal bases. Primer melting temperatures were 59–65 °C. Target fragment lengths were 80–150 bp. KASP primers were synthesized by Beijing Liuhe BGI Co.; Ltd. (Beijing, China). The AQP genotyping system was used as recommended by the manufacturer (JasonGen Biological Technology Co., Ltd.; Beijing, China). Spike length was measured to assess KASP assay efficiency for parental and F_2_ populations. Primer sequences are listed in Table S5.

### 4.5. Statistical Analysis

SPSS 26.0 software was used to analyze differences in spike length between the parents and offspring. An analysis of variance was performed using Student’s *t*-test (*p* < 0.001). GraphPad Prism 8.0 software was used to visualize data.

## 5. Conclusions

This study was completed using a long-spike mutant, *LS1*, which was obtained via EMS mutagenesis. The characteristics of this mutant included the following: dwarfism, increased spike length, increased number of spikelets, increased number of grains per spike, long grains, and improved yield per plant. By constructing a segregating population and applying BSA-seq technology, a spike length regulatory locus was mapped to a 7.22 Mb region (673.84–713.26 Mb) on chromosome 5A. This region included 12 candidate genes expressed in spikes. Additionally, two KASP markers associated with spike length were developed, both of which were applicable for genotyping the segregating population. The study findings and data provide a theoretical foundation for optimizing spike length in wheat via genetic improvement.

## Figures and Tables

**Table 2:** Statistics of SNPs and InDels location annotation results.

SNPs Mutation Region	Number of Variants	InDels Mutation Region	Number of Variants
Exonic	469,329	Exonic	35,361
Intronic	117,672	Intronic	38,173
UTR3	13,059	UTR3	4234
UTR5	10,178	UTR5	4515
Upstream	6972	Upstream	2495
Downstream	5248	Downstream	1559
Upstream; downstream	2979	Upstream; downstream	955
Splicing	925	Splicing	610
Exonic; splicing	125	Exonic; splicing	32
UTR5; UTR3	19	UTR5; UTR3	8

Note: Exonic: The mutation is located in the exon coding region. Intronic: The mutation is located in the intron region. UTR3: 3′ untranslated region. UTR5: 5′ untranslated region. Upstream: The mutation is located 2Kbp upstream of the gene. Downstream: The mutation is located 2 Kbp downstream of the gene. Upstream; downstream: The variation is located 2 Kbp upstream of the gene, and also 2 Kbp downstream of the gene. Splicing: The mutation is located at the splice site (2 bp within the intron near the exon/intron boundary). Exonic; splicing: The variation is located in the exon coding region, and simultaneously, it is also located at the splicing site (2 bp within the intron near the exon/intron boundary). UTR5; UTR3: The mutation is located in both the 5′ UTR and 3′ UTR regions of the gene.

**Table 3:** Information on the association region by the ED association analysis method.

Chrom	Start	End	Length (bp)
5A	673,837,810	674,334,747	496,938
5A	680,382,639	682,026,829	1,644,191
5A	682,106,425	682,770,497	664,073
5A	691,443,224	696,644,520	5,201,297
5A	700,302,103	701,184,634	882,532
5A	709,603,814	709,631,542	27,729
5A	711,943,509	713,264,094	1,320,586
7B	714,825,625	715,608,889	783,265
7B	717,391,811	717,688,886	297,076
7B	727,435,274	727,441,287	6014

Note: Chrom: Chromosomes of wheat, Start: Starting position of the area. End: Ending position of the area. Length (bp): Length of the region.

**Table 4:** Correlation area information statistics.

Chrom	Start	End	Length (bp)
5A	673,837,810	674,334,747	496,938
5A	680,382,639	682,026,829	1,644,191
5A	682,106,425	682,770,497	664,073
5A	691,500,001	695,000,001	3,500,001
5A	700,302,103	701,184,634	882,532
5A	709,603,814	709,631,542	27,729
5A	712,000,001	713,264,094	1,264,094
7B	714,825,625	715,608,889	783,265
7B	717,391,811	717,688,886	297,076

Note: Chrom: chromosomes of wheat. Start: Starting position of the area. End: Ending position of the area. Length (bp): Length of the region.

**Table 5:** Candidate gene information for controlling spike length in the genomic intervals.

Gene ID	Gene Function Annotation
*TraesCS5A03G1193000*	Peptidase S1, PA clan
*TraesCS5A03G1208300*	Protein kinase domain
*TraesCS5A03G1208400*	PAZ domain
*TraesCS5A03G1241100*	Protein PAIR1
*TraesCS5A03G1244300*	TIFY/JAZ family
*TraesCS5A03G1246000*	Helix-loop-helix DNA-binding domain superfamily
*TraesCS5A03G1246800*	ABC transporter A
*TraesCS5A03G1247300*	Tubulin
*TraesCS5A03G1249100*	Transcription factor GRAS
*TraesCS5A03G1250600*	Bifunctional inhibitor/plant lipid transfer protein/seed storage helical domain
*TraesCS5A03G1251200*	Bifunctional inhibitor/plant lipid transfer protein/seed storage helical domain superfamily
*TraesCS5A03G1264200*	Phosphatidylserine decarboxylase-related
*TraesCS5A03G1264300*	PLAC8 motif-containing protein
*TraesCS5A03G1266400*	Aspartic peptidase A1 family
*TraesCS5A03G1266500*	Aspartic peptidase A1 family
*TraesCS5A03G1267100*	Leucine-rich repeat
*TraesCS5A03G1267500*	Leucine-rich repeat domain superfamily
*TraesCS5A03G1268000*	RING-type E3 ubiquitin transferase,
*TraesCS5A03G1268200*	F-box domain-containing protein
*TraesCS5A03G1293500*	ACT domain
*TraesCS5A03G1304500*	ABC transporter-like
*TraesCS7B03G1190000*	Alpha/Beta hydrolase fold
*TraesCS7B03G1190400*	Lysine methyltransferase

## Data Availability

The original contributions presented in this study are included in the article/Supplementary Materials. Further inquiries can be directed to the corresponding authors.

## Supplementary Materials

The following supporting information can be downloaded at: https://www.mdpi.com/article/10.3390/plants14233703/s1, Figure S1: Phenotypic characteristics of wild-type AK58 and mutant *LS1*; Figure S2: Identification of the hot-region through the SNP-index association analysis method. Figure S3: Distribution of ED association values on chromosomes. Table S1: Information of the association regions.; Table S2: Information of the association region by the SNP-index; Table S3: Information of the association region by the ED; Table S4: Candidate SNPs loci; Table S5: KASP-labeled primers used in the experiment.
