# Supplementary material for: Identification of Spike Length Gene and Development of KASP Markers in Wheat
Source: Plants (Basel). 2025 Dec 4;14(23):3703. doi: 10.3390/plants14233703 (PMC12694121; doi:10.3390/plants14233703)
Supplement: Supplementary file 1 [file plants-14-03703-s001.zip › Supplementary Figures.pdf]

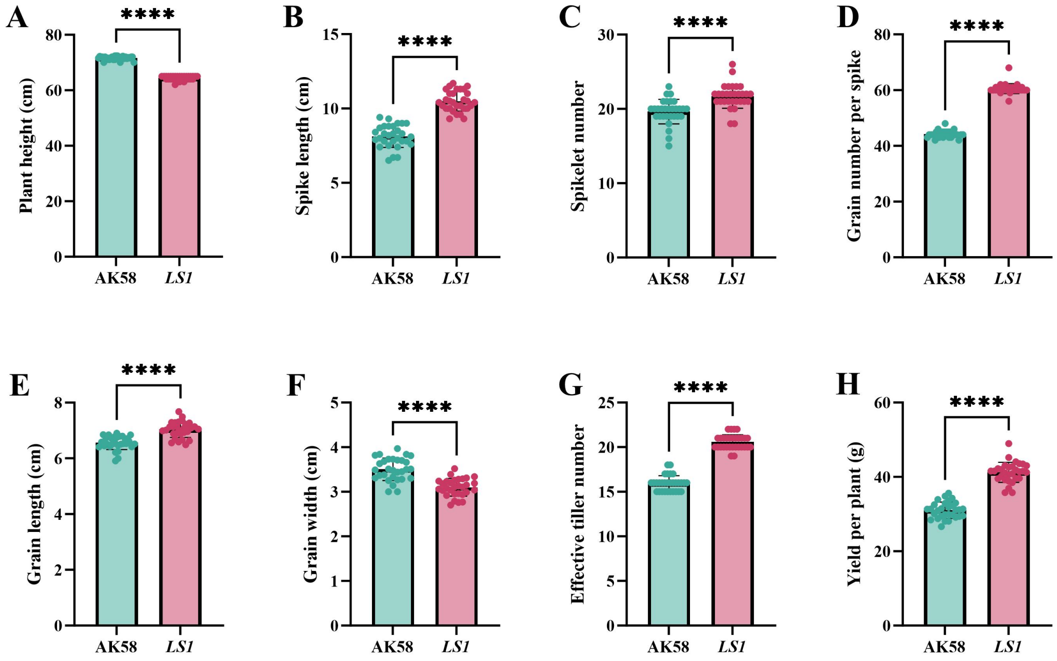

Figure S1. Phenotypic characteristics of wild-type AK58 and mutant *LS1*. Plant height (A), swpike length (B), spikelet number (C), grain number per spike (D), grain length (E), grain width (F), effective tiller number (G), and yield per plant (H) of AK58 and *LS1* . Error bar represent standard errors of three independent experiments.Asterisks indicate significant diffterences between AK58 and *LS1* according to Student's *t*-test (****$p < 0.0001$).

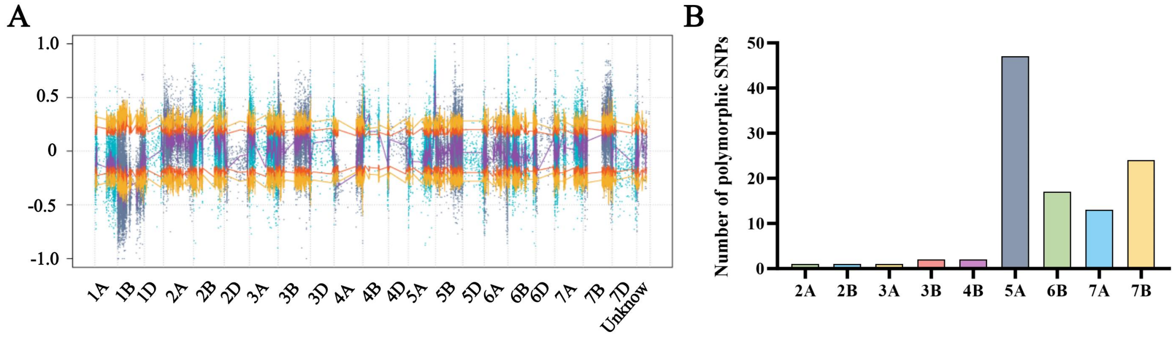

Figure S2. Identification of the hot-region through the SNP-index association analysis method. (A) Progeny Δ(SNP-index) genome distribution map. (B) The number distribution of SNPs on different chromosomes.

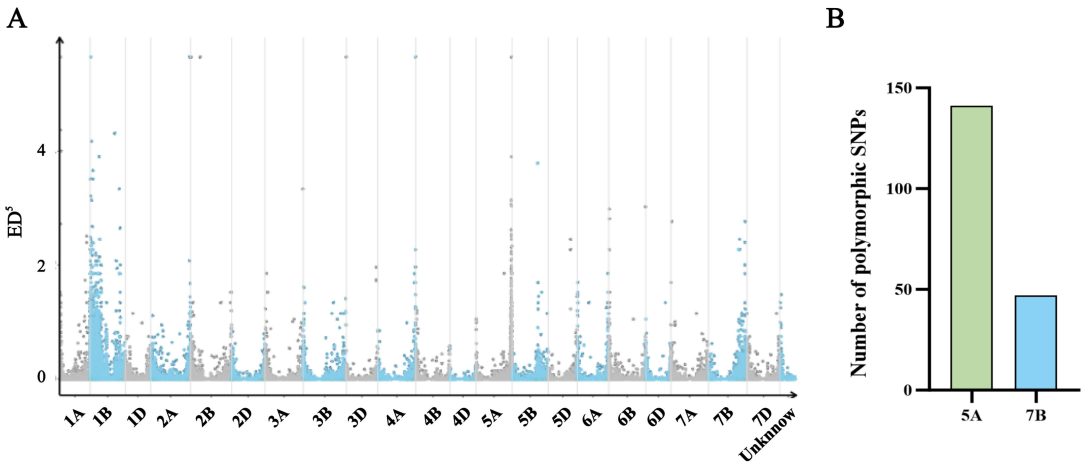

Figure S3. Distribution of ED association values on chromosomes. (A) Distribution of ED association values across the genome; (B) The number distribution of SNPs on different chromosomes.
